# Brain-Targeted Delivery of Pre-miR-29b Using Lactoferrin-Stearic Acid-Modified-Chitosan/Polyethyleneimine Polyplexes

**DOI:** 10.3390/ph13100314

**Published:** 2020-10-15

**Authors:** Patrícia Pereira, Maria Barreira, Carla Cruz, Joana Tomás, Ângelo Luís, Augusto Q. Pedro, João A. Queiroz, Fani Sousa

**Affiliations:** 1 CICS-UBI-Health Sciences Research Centre, University of Beira Interior, Avenida Infante D. Henrique, 6200-506 Covilhã, Portugal; ppereira@fcsaude.ubi.pt (P.P.); Maria_Barreira_@hotmail.com (M.B.); carlacruz@fcsaude.ubi.pt (C.C.); jtomas@fcsaude.ubi.pt (J.T.); afluis27@gmail.com (Â.L.); jqueiroz@ubi.pt (J.A.Q.); 2 Department of Chemical Engineering, Centre for Mechanical Engineering, Materials and Processes, University of Coimbra, Rua Sílvio Lima-Polo II, 3030-790 Coimbra, Portugal; 3 CICECO-Aveiro Institute of Materials, Chemistry Department, University of Aveiro, Campus Universitário de Santiago, 3810-193 Aveiro, Portugal; apedro@ua.pt

**Keywords:** blood–brain barrier, chitosan, drug delivery system, lactoferrin, polyethyleneimine, recombinant miRNA

## Abstract

The efficacy of brain therapeutics is largely hampered by the presence of the blood–brain barrier (BBB), mainly due to the failure of most (bio) pharmaceuticals to cross it. Accordingly, this study aims to develop nanocarriers for targeted delivery of recombinant precursor microRNA (pre-miR-29b), foreseeing a decrease in the expression of the BACE1 protein, with potential implications in Alzheimer’s disease (AD) treatment. Stearic acid (SA) and lactoferrin (Lf) were successfully exploited as brain-targeting ligands to modify cationic polymers (chitosan (CS) or polyethyleneimine (PEI)), and its BBB penetration behavior was evaluated. The intracellular uptake of the dual-targeting drug delivery systems by neuronal cell models, as well as the gene silencing efficiency of recombinant pre-miR-29b, was analyzed in vitro. Labeled pre-miR-29b-CS/PEI-SA-Lf systems showed very strong fluorescence in the cytoplasm and nucleus of RBE4 cells, being verified the delivery of pre-miR-29b to neuronal cells after 1 h transfection. The experiment of transport across the BBB showed that CS-SA-Lf delivered 65% of recombinant pre-miR-29b in a period of 4 h, a significantly higher transport ratio than the 42% found for PEI-SA-Lf in the same time frame. Overall, a novel procedure for the dual targeting of DDS is disclosed, opening new perspectives in nanomedicines delivery, whereby a novel drug delivery system harvests the merits and properties of the different immobilized ligands.

## 1. Introduction

Alzheimer’s disease (AD) is the most prevalent (45–60%) and devastating form of dementia in the elderly and can lead to death within 3 to 9 years after the appearance of symptoms [1]. Although several neuropathological hallmarks have been implicated in AD pathology, most data indicate that the intraneuronal amyloid plaque accumulation is constituted by aggregates of toxic amyloid-beta (Aβ) peptides play a pivotal role in the dysfunction and death of neurons [2,3,4]. Amyloid peptides are generated through sequential proteolytic cleavage of the amyloid precursor protein (APP) by β-site APP-cleaving enzyme 1 (BACE1) and γ-secretase [5,6,7]. Several convincing results suggest that the regulation of the expression of proteins involved in the generation and accumulation of Aβ peptides can be extremely important in AD since Aβ peptides are critical in the onset and development of the pathological cascade of the disease [4,5,8,9].

In recent years, several research groups demonstrated that the levels and activity of the BACE1 protein are increased in sporadic AD patients’ brains, suggesting that BACE1 dysregulation is directly implicated in AD pathogenesis. For these reasons, BACE1 has been recognized as a promising drug target for the therapy of this disease, once BACE1 inhibition may decrease the formation of all forms of Aβ peptides and, consequently, reduce cell death [10,11,12]. On the other hand, as a result of the relatively recent and extensive research, it is already clear that microRNAs (miRNAs) often display a brain-specific expression pattern [13] and can play important regulatory roles in the molecular control of a variety of neurobiological functions [14,15,16]. Until now, 18 clinical trials involving neurodegenerative diseases and miRNAs have been documented in the database of the National Institute of Health based in the USA (www.clinicaltrials.gov), which strongly suggests the high potential for miRNAs to be used as biopharmaceuticals or as biomarkers, respectively, in the treatment or diagnosis of neurodegenerative disorders [14,17]. However, the manufacturing (production, recovery, and purification) and delivery of miRNAs into the brain poses many technical challenges, which need to be addressed if the application of these molecules in pre-clinical and clinical trials is envisaged [18]. Recently, our research group proposed an innovative strategy that allows the biosynthesis and purification of recombinant pre-miR-29b to decrease the BACE1 and endogenous Aβ peptides expression levels [19]. Regarding the delivery of therapeutic agents for the treatment of brain diseases, it is well known that this is intrinsically limited because the delivery of all substances into the brain is tightly regulated by the blood–brain barrier (BBB) [18]. The BBB is a physical barrier that restricts the transport of a large number of therapeutic drugs (with the exception of certain nutrients, namely amino acids and neuropeptides) from the circulating blood to the brain [18,20,21,22,23], making the brain a site of poor permeability to various drugs as well as drug delivery systems (DDSs), which can limit treatment efficacy. Indeed, the BBB displays a variety of efflux transporters, including P-glycoprotein [24], receptors for transferrin [25,26], lactoferrin (Lf) [27], lipoprotein receptor-related protein (LRP) [28,29], among others, which reinforce its ability to effectively remove drugs from the brain by pumping them back into the blood. In this way, the receptors existing on the BBB have been explored to target drugs to the brain specifically, thus facilitating the ability of a drug containing a specific ligand to interact with its specific receptor expressed on the luminal side of BBB endothelial cells.

During the last decade, the use of polymeric nanoparticles (e.g., based on polyethyleneimine (PEI), chitosan (CS), poly (lactic-*co*-glycolic acid) (PLGA), polylactic acid (PLA)), polymeric micelles, liposomes, and dendrimers modified with biological ligands has emerged as one of the most attractive approaches to enhance targeting to the brain [18]. Chitosan (CS) and polyethyleneimine (PEI) have gained much attention as DDSs due to their advantageous properties, such as the ability to encapsulate large amounts of the drug, high stability in biological fluids, targeting ability, rapid cellular uptake, high transfection efficiency, biocompatibility, biodegradability, low cytotoxicity and immunogenicity, and reduced side effects by targeted delivery [30,31,32]. In addition, the cationic charge of CS and PEI, due to the presence of amino groups, allows the establishment of electrostatic interactions with negatively charged RNAs, leading to an effective condensation and protection of RNA. In fact, the encapsulation of RNAs prevents non-specific interactions and enzymatic degradation, thereby increasing the drug circulation in blood [30,31,32]. Previously, our research group has reported the preparation and characterization of CS and PEI polyplexes to deliver pre-miR-29 in neuronal cells [19]. However, it is essential to develop novel DDSs that enable the targeted delivery of nanomedicines to the brain in a more effective and safer way.

Lf is an iron-binding glycoprotein (80 kDa), with many biological functions, such as anti-bacterial, anti-inflammatory, anti-viral, immunomodulatory, antioxidant, and anti-tumor properties [33,34]. The lactoferrin receptors are highly expressed in brain endothelial cells and neurons [35,36], and recently, Lf was described as a neuroprotective agent, being thus a valuable therapeutic candidate for the treatment of neurodegenerative diseases [37,38,39]. Wang and collaborators reported that internalized exogenous Lf promoted the non-amyloidogenic α-secretase processing of APP by activating the extracellular signal-regulated kinases (ERK)-cAMP response element-binding protein (CREB) and hypoxia-inducible factor 1-alpha (HIF-1α) signaling pathways in APP/PS1 mice and N2aSW cells, which consequently reduced Aβ aggregation and improved cognitive learning ability in APP/PS1 mice [40]. These findings provide mechanistic insight into the potential use of Lf as a valuable therapeutic agent for the treatment of AD, on the one hand, being itself a promising brain-targeting ligand for the modification of cationic polymeric systems [41,42], facilitating drug delivery into the brain. In turn, stearic acid (SA) is an endogenous long-chain saturated fatty acid, which presents low toxicity and substantial biocompatibility and offers substantial drug loading [43]. Moreover, the CS-SA copolymer has already been used to deliver drugs into the brain, showing excellent membrane penetration ability, which increased in vitro cellular uptake and enhanced the therapeutic effect [44]. Additionally, it has been confirmed that CS-SA had the ability to open transient biobarriers and bypass the P-gp system (avoiding recognition by P-gp), yielding an efficient brain-targeting gene vector [44].

Taking this into account, the current work envisioned the dual conjugation of SA and Lf with CS and PEI, to combine the merits of all polymers/ligands, aiming to achieve an effective and safe ligand-mediated brain-targeted DDS for a recombinant pre-miRNA. Thus, the following modified polyplexes were prepared: SA-modified PEI and SA-modified CS (PEI-SA and CS-SA, respectively), and SA and Lf-modified PEI and SA and Lf-modified CS (PEI-SA-Lf and CS-SA-Lf, respectively). These nanoparticles were characterized, and their structure confirmed using distinct techniques. Moreover, the properties of the prepared polyplexes, namely their ability to transport across the BBB model, the targeting effects, in vitro cell uptake, and transfection studies were evaluated on N2a695 and RBE4 in vitro models. The efficacy of these DDSs was finally monitored by examining the induced BACE1 inhibition.

## 2. Results and Discussion

### 2.1. Preparation and Structural Characterization of CS/PEI-SA and CS/PEI-SA-Lf

**Figure 1:**
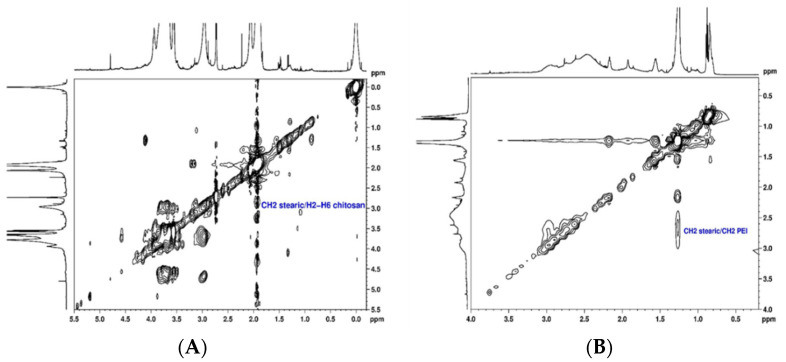
Structural characterization of the chitosan–stearic acid (CS-SA) and polyethyleneimine–stearic acid (PEI-SA) conjugates: (**A**) ^1^H, ^1^H-TOCSY spectrum of stearic acid bound to chitosan in D_2_O; (**B**) ^1^H, ^1^H-NOESY spectrum of stearic acid bound to polyethyleneimine in CDCl_3_.

The conjugation of SA to CS and PEI polymers (CS-SA and PEI-SA conjugates) was performed by a chemical reaction between the carboxyl group of SA and the amino group of CS or PEI in the presence of 1-ethyl-3-(3-dimethylaminopropyl) carbodiimide (EDC)/N-Hydroxysuccinimide (NHS), as a carboxyl activating agent [44,45]. The structural characterization of the CS-SA and PEI-SA conjugates were performed by NMR spectroscopy and the 2D spectra are presented in Figure 1. The ^1^H NMR spectra of the CS and PEI attached to SA are shown in Supplementary Material (Figure S1 and S2, respectively). The chemical shift (δ) was expressed as parts per million (ppm). In the ^1^H NMR spectrum of CS-SA conjugate, the upfield resonances at δ = 0.98 ppm and δ = 1.1–1.2 ppm could be attributed to the methyl and methylene’s protons of the stearate group, respectively [44]. Furthermore, the coupling between the stearate group and the polymer was identified in the TOCSY spectrum (Figure 1A) by the cross-correlation peaks between methylene’s protons from the stearate group at 1.1 ppm and H2-H6 (Figure S3 of Supplementary Material) at δ ~3.6 ppm of CS. These results prove that SA was successfully grafted onto CS. Similarly, the following chemical shifts were observed in the ^1^H NMR spectrum of the PEI-SA conjugate (Figure S2 of Supplementary Material): a sharp peak at δ = 1.26 ppm corresponding to 28 methylene protons of stearate group, two peaks between δ = 2.17 ppm and δ = 1.93 ppm corresponding to two CH_2_ adjacent to the amide group and one peak at δ = 0.85 ppm attributed to the methyl group. From the NOESY spectrum (Figure 1B), a cross-correlation peak between the protons CH_2_ of the stearate group at δ = 2.17 ppm and the methylene resonances from the PEI at δ = 2.50–2.86 ppm is also noticeable, which confirmed the successful modification of PEI-SA. These findings prove that the coupling of the stearate group to the CS and PEI polymer could be achieved via an EDC-mediated reaction [44,46]. Afterward, for the modification of the system with Lf, Lf was first thiolated using 2-iminothiolane hydrochloride (2-IT) as the sulfhydrylization reagent, and thiolated Lf was obtained after purification. The coupling of Lf to CS-SA was identified in the ^1^H NMR spectrum (Figure S4 of Supplementary Material) by the disappearance of the stearate peaks at δ = 0.98 ppm and δ = 1.1–1.2 ppm, indicating that the stearate group had reacted with the thiol group of Lf. In turn, it was also deduced from the spectrum of PEI-SA-Lf conjugates (Figure S5 of Supplementary Material) that the methylene protons at δ = 1.25 ppm and the methyl group at δ = 0.87 ppm were not detected, indicating that the stearic group had reacted with the thiol group of Lf. In addition, the conjugation of Lf to CS-SA and PEI-SA was also confirmed by SDS-PAGE, where a new band above 80 kDa was observed, indicating that Lf ligand was covalently attached to the CS/PEI-SA conjugates (Figure S6 of Supplementary Material) [47].

### 2.2. Polyplexes Characterization

**Figure 2:**
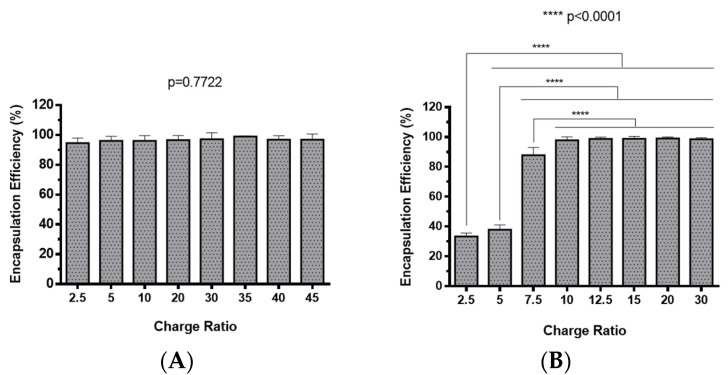
Encapsulation efficiency of different pre-miR-29b-loaded polyplexes at various N/P ratios: (**A**) CS-SA/pre-miR-29b polyplexes and (**B**) PEI-SA/pre-miR-29b polyplexes.

**Figure 3:**
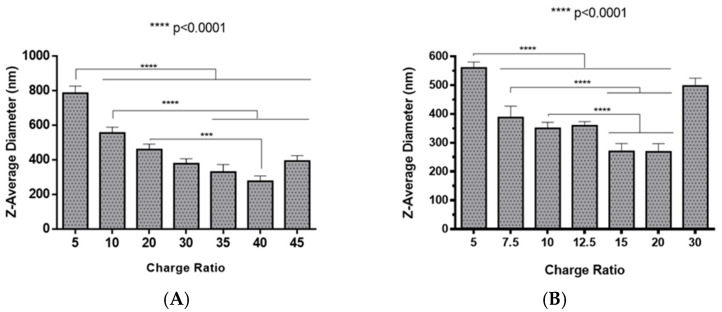
Z-average diameter of different pre-miR-29b-loaded polyplexes at various N/P ratios in distilled water: (**A**) CS-SA/pre-miR-29b polyplexes and (**B**) PEI-SA/pre-miR-29b polyplexes.

**Figure 4:**
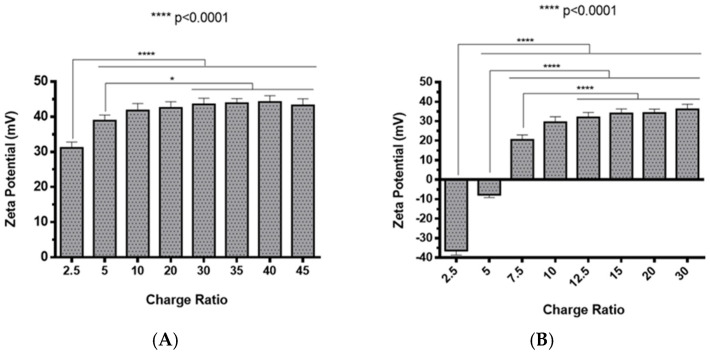
Zeta potential of different pre-miR-29b-loaded polyplexes at various N/P ratios in distilled water: (**A**) CS-SA/pre-miR-29b polyplexes and (**B**) PEI-SA/pre-miR-29b polyplexes.

The preparation of polyplexes was based on the formulation previously optimized by our research group [48], which relies on the establishment of electrostatic interactions between positively charged amine groups of the prepared conjugates with negatively charged phosphate groups from RNA. Polyplexes were prepared at different N/P ratios (ratio of positively-chargeable polymer amine (N, nitrogen) groups to negatively-charged nucleic acid phosphate (P) groups), and a complete characterization was performed, including the determination of encapsulation efficiency (EE), z-average diameter and polydispersity index (PDI), as well as the zeta potential. These parameters are critical aspects influencing cell transfection and in vitro activity of encapsulating nanomedicines. Initially, a UV spectrophotometry assay (Figure 2) and agarose gel electrophoresis analysis (Figure S7 of Supplementary Material) were conducted to determine the EE of pre-miR-29b structure when conjugated with the prepared systems. These results demonstrated that the EE of the CS-SA/RNA polyplexes was more than 95% at all N/P ratios (Figure 2A) and was 96% for the 35 N/P ratio when using the polymer conjugated with Lf (CS-SA-Lf/RNA) (see Table 1), indicating that pre-miR-29b was almost completely complexed with CS-SA and CS-SA-Lf conjugates. In the case of PEI-SA conjugates, the effective pre-miR-29b complexation was noticed at N/P ratios greater than 10, as verified by the increase in the EE (Figure 2B); here, circa 97% of RNA was complexed with PEI-SA conjugates, and an increased EE was exhibited as the N/P ratio increased (Figure 2B). Similar to the results obtained with CS, the EE of the PEI-SA/RNA polyplexes was more than 97% for the 10–30 N/P ratios (Figure 2B) and ~96% when using the conjugation with Lf (PEI-SA-Lf/RNA) at 15 N/P ratio (Table 1). The RNA condensation efficiency of prepared conjugates formed at different N/P ratios was also confirmed by agarose gel retardation assay (Figure S7 of Supplementary Material). As shown in Figure S7 of Supplementary Material, the EE of RNA was N/P ratio-dependent for PEI-SA conjugates, which was consistent with the UV spectrophotometry analysis. In the case of the CS-SA conjugates, the EE of RNA was not N/P ratio-dependent due to the influence of the size of the polymer in the polyanion compaction [48]. After completing the EE study, the z-average diameter of the formed polyplexes at various charge ratios was determined, as shown in Figure 3. The size measurements achieved by dynamic light scattering revealed that all prepared conjugates were able to efficiently condense RNA in monodisperse particles with size ranging from 270 nm up to 700 nm (Figure 3). At 35 N/P ratio, the z-average diameter of the CS-SA/RNA polyplexes dispersed in water was ~329 nm (Figure 3A), whereas CS-SA-Lf/RNA polyplexes (Table 1) presented a z-average diameter of ~326 nm (Table 1). In turn, at 15 N/P ratio, the PEI-SA/RNA polyplexes dispersed in water showed a z-average diameter of ~270 nm (Figure 3B), and it changed to about 290 nm for PEI-SA-Lf/RNA polyplexes (Table 1). Overall, the z-average diameters of the CS-based polyplexes were slightly higher than those obtained with PEI-based polyplexes to the different N/P ratios, which can be explained by the fact that CS is also a larger polymer [48]. Moreover, Dynamic Light Scattering (DLS) analysis showed that the z-average diameters of SA-Lf-modified polyplexes increased compared with that of SA-modified polyplexes because of the conjugation with Lf ligand, which is in good agreement with previous works [49,50]. Figure 4 depicts the zeta potential measurements of prepared polyplexes at different N/P ratios. The zeta potential analysis revealed that the CS-SA/pre-miR-29b polyplexes always exhibited positive surface charges in all N/P ratios, with values ranging from +31 to +44 mV (Figure 4A). At 35 N/P ratio, CS-SA/pre-miR-29b polyplexes exhibited a zeta potential value of +44 mV, while the zeta potential value for CS-SA-Lf/pre-miR-29b was +33 mV, indicating that the conjugation with Lf influences the surface charge of polyplexes (Table 1). As shown in Figure 4B, a change from negative to positive zeta potential values of PEI-SA polyplexes was observed as the N/P ratio increased from 2.5 to 7.5 (ranging between -36 and +20 mV). These findings are well in agreement with the data from the UV assays (Figure 2B) and agarose gel electrophoresis (Figure S7B of Supplementary Material), indicating that, at this point, RNA was not fully complexed with the CS-SA polycation. For high N/P ratios (10 to 30), the PEI-SA/pre-miR-29b polyplexes always presented positive surface charges (+29 to +36 mV). The zeta potential values of PEI-SA polyplexes were N/P ratio-dependent once an increase in the concentration of polymer at a constant RNA concentration led to an increased value of zeta potential of the polyplexes until it reached a plateau with no significant variations. At 15 N/P ratio, when the PEI-SA conjugate was modified with Lf, the zeta potential was slightly lower (+11 mV) when compared with values of zeta potential of the PEI-SA/pre-miR-29b polyplexes without Lf (+34 mV). The results showed that the zeta potential values of the CS-SA and PEI-SA polymers with the conjugation of Lf on the surface were decreased, what could be explained by the modification of the surface of the polymers with Lf causing a decrease in the number of protonated amino groups on polymers, leading to the drop of zeta potential. As reported in the literature, the use of positively charged polyplexes can enhance their interaction with the negatively charged cell membrane by electrostatic interactions, thus enabling the polyplexes to enter the cell and simultaneously preventing their aggregation [48]. The positive charges can additionally be involved in the destabilization of the endosomal membrane, thereby causing the release of the polyplexes into the cytosol and an increased biological activity [18]. Finally, SEM images showed that prepared polyplexes displayed a very well defined spherical and uniform morphology (Figure S8 of Supplementary Material), which is also an important feature for cell transfection.

### 2.3. Polyplexes Cytotoxic Profile

**Figure 5:**
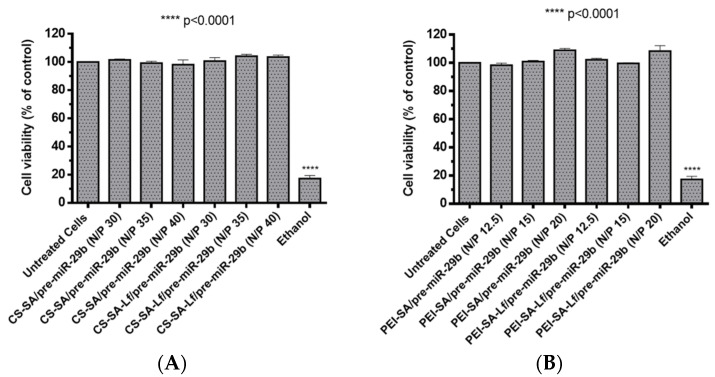
Relative cell viability of RBE4 cells treated with different pre-miR-29b-loaded polyplexes at various N/P ratios for 24 h: (**A**) CS-based polyplexes and (**B**) PEI-based polyplexes.

Foreseeing the evaluation of the biocompatible character of prepared polyplexes, two different cell types (rat brain microvascular endothelial (RBE4) and mouse neuroblastoma cells stably transfected with cDNA encoding human APP695 (N2a695) cell lines) were treated with suspensions containing polyplexes at different N/P ratios (30, 35, 40 N/P ratio for CS-based polyplexes and 12.5, 15, 20 N/P ratio for PEI-based polyplexes). These N/P ratios were selected based on the results obtained from the calculations of EE, zeta potential, and size determinations. The cytotoxicity was evaluated by an in vitro MTS (3-(4,5-dimethylthiazol-2-yl)-5-(3-carboxymethoxyphenyl)-2-(4-sulfophenyl)-2H-tetrazolium) assay. N2a695 cells were used because they present a constitutive endogenous hBACE1 expression, therefore, enabling greater sensitivity for detecting pre-miR-29b induced changes in the hBACE1 expression at the post-transcriptional level [51]. Additionally, several studies have demonstrated that the neuroblastoma cell line expresses the lactoferrin receptor [40,52,53], thus representing a very good model to investigate the targeting ability of the formulated polyplexes. In turn, RBE4 cells are considered in vitro models of the rat BBB because they express all the enzymes and transporters that are considered to be specific of the BBB endothelium, with similar characteristics to those expected from in vivo analysis [54,55,56]. RBE4 cell monolayers have also been used to investigate the mechanism of the transendothelial transport of large molecules, such as nanoparticles, with potential application as drug delivery vectors to the brain [54,55]. As shown in Figure 5 and for an incubation period of 24 h, the prepared polyplexes did not induce any significant cytotoxicity (cell viability was above 98%) for RBE4 cells. Similarly, for N2a695 cells and an incubation period of 72 h, the prepared polyplexes did not show any appreciable cytotoxicity (cell viability was over 93%) in the same N/P ratios (Figure S9 of Supplementary Material). In general, these results indicate that the difference in the cellular viability between conjugates with SA and conjugates with SA-Lf was not significant and that all prepared polyplexes were additionally identified as non-cytotoxic, demonstrating high biocompatibility, therefore, being suitable for therapeutic applications and the in vitro transport across the BBB.

### 2.4. In Vitro Cellular Uptake and Intracellular Distribution of Pre-miR-29b-Loaded Complexes

**Figure 6:**
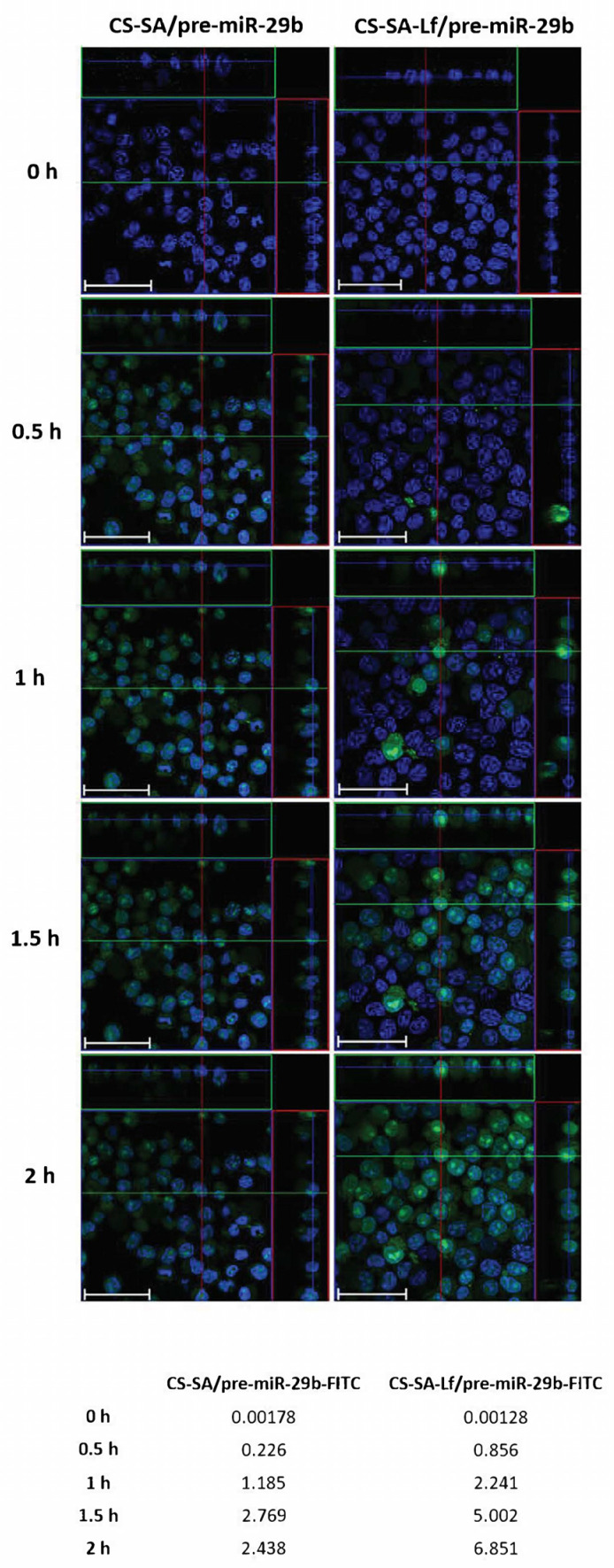
Confocal laser scanning microscopy imaging of N2a695 cells treated with CS-SA/pre-miR-29b-fluorescein isothiocyanate isomer I (FITC) (35 N/P ratio) and CS-SA-Lf/pre-miR-29b-FITC (35 N/P ratio) polyplexes after different periods of incubation (0.5, 1, 1.5, and 2 h). The fluorescence signals were collected by Laser Scanning Confocal Microscopy (LSCM) with three channels: blue fluorescence from nuclei stained of cells with Hoechst 33342^®^ (blue), green fluorescence from FITC labeled pre-miR-29b, and the merged images of three channels. Representative immunostaining data showing most of the FITC-labelled CS-SA-Lf/pre-miR-29b polyplexes localized in the cytoplasm. Scale bars 50 μm. The values are relative to the average FITC fluorescence intensity.

Based on the initial results from the EE, zeta potential, particle size, and cytotoxicity of the polyplexes, an N/P ratio of 35 both for CS-SA and CS-SA-Lf conjugates, and an N/P ratio of 15 for PEI-SA and PEI-SA-Lf conjugates were chosen for further in vitro experiments, thereby ensuring an overall positive charge and effective interaction of the polyplexes with a negatively charged cell membrane. To evaluate the intracellular uptake and distribution of pre-miR-29b loaded onto the CS-SA, CS-SA-Lf, PEI-SA, and PEI-SA-Lf conjugates, confocal laser scanning microscopy was employed (Figure 6 and Figure S10 of Supplementary Material). This was accomplished by labeling the pre-miR-29b with fluorescein isothiocyanate isomer I (FITC), thereby allowing for tracking polyplexes fluorescence and inferring their localization in N2a695 cells. Figure 6 presents the confocal fluorescence microphotographs of N2a695 cells, used as a model of brain cells, treated with FITC-labeled CS-SA/pre-miR-29b and CS-SA-Lf/pre-miR-29b after 0, 0.5, 1, 1.5, and 2 h incubation. In the image, green fluorescent (FITC) corresponds to pre-miR-29b-labeled polyplexes and the blue fluorescence to cell nuclei stained by Hoechst 33342^®^. In general, the fluorescence intensity inside cells increased gradually, and a significant fluorescence was observed after 2 h incubation (Figure S10 of Supplementary Material). The serial Z-stacks of confocal images showed a diffuse distribution of CS-SA-Lf/pre-miR-29b-FITC polyplexes within the cells, with a significant proportion of polyplexes localized primarily in the cytoplasm near the cell nucleus, after 0.5 h of incubation (Figure 6). For longer incubation periods, pre-miR-29b-loaded complexes were also present in the nuclei of cells, a strong green fluorescence being visible throughout the entire cytoplasm and nuclei of N2a695 cells. The fluorescence intensity of CS-SA-Lf/pre-miR-29b-FITC polyplexes was higher than the fluorescence obtained for cells treated with CS-SA/pre-miR-29b-FITC polyplexes, demonstrating that the Lf surface modification increased cellular uptake of the polyplexes (Figure S10 of Supplementary Material). In contrast, the mean fluorescence intensity of the PEI-SA/pre-miR-29b polyplexes increased during the 2 h of incubation, while PEI-SA-Lf/pre-miR-29b increased until 1.5 h of incubation and then remained stable until the end of the transfection period (data not shown). These results indicate that the release of pre-miR-29b from the CS-SA-Lf complexes was faster than that from the PEI-SA-Lf. Besides, these findings demonstrated that the manufactured polyplexes displayed an excellent ability to cross cell membranes and deliver pre-miR-29b efficiently to N2a695 neuronal cells. Thus, these results clearly demonstrate the effect of targeting-ligands, since the polyplexes modified with Lf ligand had faster uptake compared to the polyplexes that were unmodified by the target ligand, suggesting that cellular uptake occurs due lactoferrin receptor-mediated endocytosis. These data can be explained because the neuroblastoma cells contain lactoferrin receptors on the surface [40,51,52,53,57].

### 2.5. Human BACE1 Gene Knockdown Using Pre-miR-29b-Loaded Complexes

**Figure 7:**
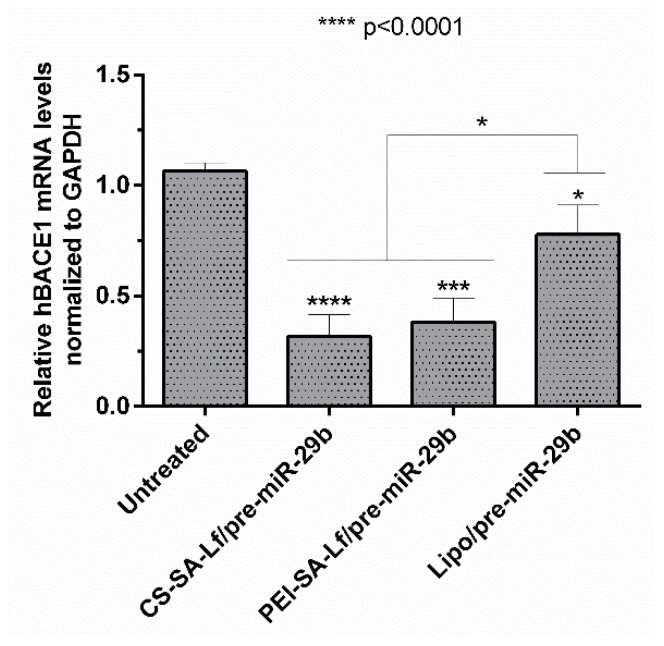
In vitro gene silencing effect of recombinant pre-miR-29b on hBACE1 mRNA levels in N2a695 cells after 72 h treatment with CS-SA-Lf/pre-miR-29b (35 N/P ratio), PEI-SA-Lf/pre-miR-29b (15 N/P ratio) and Lipofectamine 2000 (Lipo)/pre-miR-29b. Values in the graph represent the mean from triplicates of RT-qPCR threshold cycles for hBACE1 mRNA normalized to those of mRNA for GAPDH from three independent experiments and demonstrate significant differences across treatment conditions.

BACE1 gene knockdown efficiency induced by recombinant pre-miR-29b was investigated using N2a695 cells transfected with the prepared conjugates (35 N/P ratio CS-SA-Lf and 15 N/P ratio PEI-SA-Lf) and Lipofectamine 2000 (Lipo) containing 10 nM of the target pre-miRNA, for comparison. The expression of the BACE1 mRNA levels was evaluated by RT-qPCR and normalized to the GAPDH level. Figure 7 shows that the levels of hBACE1 mRNA were reduced after 72 h of transfection to approximately 68% in cells transfected with CS-SA-Lf/pre-miR-29b, and by 60% in those transfected with PEI-SA-Lf/pre-miR-29b complexes, compared to the cells without transfection. This reduction in mRNA hBACE1 expression was also significantly higher than the silencing (around 22%) achieved in cells transfected with Lipo/pre-miR-29b, the positive control (Figure 7). Together, these results demonstrate the enhanced performance displayed by CS-SA-Lf over Lipo in the delivery of pre-miR-29b to neuronal cells.

### 2.6. Ability of Prepared Polyplexes to Cross in an In Vitro BBB Model

**Figure 8:**
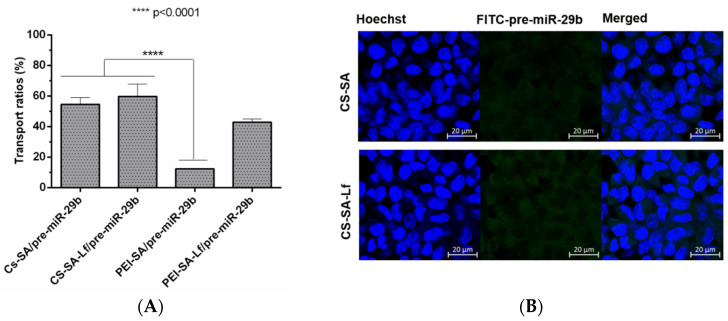
The transport ratio of pre-miR-29b across the blood–brain barrier BBB during 4 h in RBE4 cells. (**A**) Intracellular cells after treating with CS-SA/pre-miR-29b-FITC (35 N/P ratio), CS-SA-Lf/pre-miR-29b-FITC (35 N/P ratio), PEI-SA/pre-miR-29b-FITC (15 N/P ratio), and PEI-SA-Lf/pre-miR-29b-FITC (15 N/P ratio). Quantification of fluorescence intensity for FITC was performed in the fluorimeter. Error bars represent standard deviations derived from three or more independent experiments performed in triplicate. ANOVA, mean ± SD. (**B**) Representative confocal microscopy images of RBE4 cells cultured in the transwells treated with CS-SA/pre-miR-29b-FITC and PEI-SA-LF/pre-miR-29b-FITC. The fluorescence signals were collected by LSCM with three channels: blue fluorescence from nuclei stained with 33342^®^ (blue), green fluorescence from FITC labeled pre-miR-29b and the merged images of three channels. Scale bars 20 μm.

Permeabilization experiments foreseeing the analysis of the ability of the prepared polyplexes to cross the BBB were conducted using an in vitro BBB model based on RBE4 cells. To further confirm that RBE4 effectively mimic the BBB, experiments were performed under a transendothelial electrical resistance (TEER) value above 250 Ωcm^2^ [58]. The fluorescence intensity of the polyplexes (CS-SA/pre-miR-29b-FITC, CS-SA-Lf/pre-miR-29b-FITC, PEI-SA/pre-miR-29b-FITC, and PEI-SA-Lf/pre-miR-29b-FITC) in the inner and outer chambers of the transwell was determined to evaluate if conjugation of CS and PEI polymers with Lf and SA effectively leads to an increased ability of polyplexes to cross the BBB. Figure 8A shows the results of the transportation ability of different polyplexes across the BBB model using the same concentration of pre-miR-29b. After 4 h of incubation, the different polyplexes were internalized by RBE4 cells in the following order: CS-SA-Lf/pre-miR-29b > CS-SA/pre-miR-29b > PEI-SA-Lf/pre-miR-29b > PEI-SA/pre-miR-29b, with transportation ratios of 59.68%, 54.58%, 42.89%, and 12.29%, respectively. These findings clearly demonstrate that the Lf ligand is responsible for a remarkable increase in the ability of polyplexes to cross the BBB, as the dual-targeting polyplexes (PEI-SA-Lf and CS-SA-Lf) have a greater transportation ability to cross the BBB compared to the complexes with only one targeting group (PEI-SA and CS-SA). Several studies have demonstrated that Lf can assist in nanoparticle-based DDSs through the BBB [59,60,61]. Figure 8B shows the immunostaining microphotographs of CS-SA-Lf and PEI-SA-Lf internalized in RBE4 cells. As revealed in Figure 8B, the strong green intensity in the cytoplasm of RBE4 cells suggested that complexes were internalized by the cells. These results could be explained by the fact that Lf ligand mediated the recognition via lactoferrin receptors expressed on the surface of RBE4 cells, allowing the transport of exogenous pre-miR-29b into the brain, probably via the pathway of receptor-mediated transcytosis (RMT) [59,60,61,62]. On the other hand, these results can also be explained due to the positive charge of the cationic polymers, which could promote electrostatic interactions with the negatively charged RBE4 cell membranes, activating the cellular uptake through the pathway of adsorptive-mediated transcytosis (AMT) [44,63]. As indicated, the internalization of the polyplexes of CS by the RBE4 cells was higher than that of PEI polyplexes. These results can be related to some characteristics of CS polymer that can transiently open tight junctions, enabling the transport of components via a paracellular pathway through the epithelial barrier [32].

Overall, the delivery vehicles herein described are endowed with enhanced performance; therefore, being responsible for a more targeted and efficient pre-miR-29b delivery and are able to cross the BBB.

## 3. Materials and Methods

### 3.1. Chemicals and Reagents

Arginine–Sepharose 4B gel was acquired from GE Healthcare Biosciences (Uppsala, Sweden). All buffers used for the chromatographic experiments were freshly prepared with 0.05% diethyl pyrocarbonate (DEPC) treated water and were filtered through a 0.20 µm pore sized membrane. Sodium chloride (NaCl) was purchased from Panreac and tris (hydroxymethyl) aminomethane (Tris) from Sigma–Aldrich. PEI of M*w* 2 kDa, CS medium molecular weight (M*w* 190–310 kDa; degree of deacetylation in the 75–85% range), SA Grade I (98.5%), human lactoferrin, 2-iminothiolane hydrochloride (2-IOT, 98%), 1-ethyl-3-(3-dimethylaminopropyl) carbodiimide (EDC), *N*-Hydroxysuccinimide (NHS) and all cell culture media and reagents used in cell culture procedures were acquired from Sigma-Aldrich (St. Louis, MO, USA). Fluorescein isothiocyanate isomer I (FITC), Hoechst 33342^®^, and AlexaFluor 488^®^ were obtained from Invitrogen (Carlsbad, CA, USA). Mouse neuroblastoma cells stably transfected with cDNA encoding human APP695 (N2a695) were kindly provided by Professor Wenjie Luo (Weill Cornell Medical College). The rat brain microvascular endothelial cell line (RBE4) was provided as a gift by the laboratory of Dr. M. Aschner (Department of Pediatrics, Vanderbilt Kennedy Center, Nashville, TN, USA).

### 3.2. Synthesis of CS and PEI Conjugated with SA

The conjugation of CS or PEI to SA (designated as CS-SA and PEI-SA, respectively) was performed as previously reported by Xie and co-workers [44], with modifications. In brief, first, SA (2.5 mg) and EDC/NHS (25 mg, at a ratio 1:10) were dissolved in 1.0 mL anhydrous DMSO and stirred at 60 °C for 1 h, until EDC and SA were well-dissolved and mixed. The resulting mixture (SA:EDC) was then added slowly to 1% (*w/v*) of CS and PEI in sodium acetate buffer (0.1 M sodium acetate/0.1 M acetic acid, pH 4.5), and the reaction solution was kept at 25 °C in the dark for 24 h, with stirring in a water bath. In the presence of EDC, the amino groups on the surface of polymers specifically reacted with the carboxyl groups of SA. Posteriorly, the resulting conjugate, CS/PEI-SA, was dialyzed first against phosphate-buffered saline (PBS, pH 7.4) for 1 day and then against double deionized water for 2 days, using a dialysis membrane (SnakeSkin^TM^ Dialysis Tubing, MWCO 3.5 kDa, 22 mm dry diameter, ThermoScientific). The conjugated CS/PEI-SA was isolated as a “*sponge*” by lyophilization and was further characterized by nuclear magnetic resonance (NMR) spectroscopy.

### 3.3. Functionalization of CS/PEI-SA with Lactoferrin

The functionalization with Lf was obtained using a common method with 2-iminothiolane hydrochloride as the sulfhydrylization reagent [49]. For the preparation of CS/PEI-SA-Lf, Lf (10 mg) was dissolved in 1.0 mL aqueous solution of water and mixed with 1.0 mL aqueous solution of 2-iminothiolane hydrochloride (2-IOT, 0.7 mg), and the reaction proceeded for 1 h at room temperature, with moderate shaking. The excess of 2-IOT was removed by gel filtration chromatography using Sephadex G 100 (Hitrap desalting column) and with PBS 1× (pH 7.0) as the mobile phase. Fractions were collected according to the chromatogram obtained by analysis of the absorbance (a peak contains about 90% of thiolated Lf) [49]. Subsequently, CS/PEI-SA was dissolved in sodium acetate buffer (pH 4.5) and then added to the PBS solution of Lf, drop by drop, followed by incubation for 20 h at room temperature, in the dark. To remove unreacted Lf, the resulting mixtures were purified by dialysis (SnakeSkin^TM^ Dialysis Tubing, MWCO 3.5 kDa, 22 mm dry diameter, ThermoScientific) during 4 days against double deionized water and freeze-dried, for further usage. NMR spectroscopy and SDS-PAGE were used to confirm further the surface capping of the terminal amines of the CS/PEI-SA with Lf.

### 3.4. NMR Experiments

All NMR experiments were performed at room temperature using a Bruker Avance III 600 operating at 600.10 MHz for protons, equipped with a QCI cryoprobe. All spectra were acquired under field-frequency locked conditions using the probe channel with the spectrometer’s lock hardware. Spectra were processed using Bruker Topspin 3.2. All ^1^H NMR spectra were referenced internally to the trimethylsilyl propionate signal in D_2_O and tetramethylsilane in CDCl_3_. Approximately 10 mg of sample (CS-SA, PEI-SA, CS-SA-Lf, and PEI-SA-Lf) was weighed and placed in a 5-mm NMR tube (600 μL). Water suppression pulse using excitation sculpting with gradients (*zgesgp*) was used to reduce the 1H signals of water. NOESY experiments were acquired with 300 ms mixing time in 16 transients with a relaxation delay of 3.0 s and a spectral width of *ca* 6000 Hz in a total of 2 K data points in F2 and 256 data points in F1. 2D TOCSY experiments were acquired by DIPSI2 sequence with 80 ms mixing time in 16 transients with a relaxation delay of 3.0 s and a spectral width of ca. 4000 Hz, in a total of 2 K data points in F2 and 256 data points in F1.

### 3.5. Preparation of Polymer-RNA Complexes (Polyplexes)

The human pre-miR-29b used in the experiments was obtained from a bacterial cell culture of *Rhodovulum sulfidophilum* DSM 1374 strain (BCCM/LMG, Belgium) modified with the plasmid pBHSR1-RM containing the sequence of pre-miR-29b [64]. After biosynthesis, the recombinant pre-miR-29b was recovered from a complex mixture of small RNAs and purified by arginine-affinity chromatography, as we previously described [65]. The quality of the purified pre-miR-29b was assessed by polyacrylamide electrophoresis. CS-SA, PEI-SA, CS-SA-Lf, and PEI-SA-Lf systems were dissolved in sodium acetate buffer (concentration of 10 mg/mL) and mixed with 10 nM of recombinant pre-miR-29b at different polymer/RNA (N/P) charge ratio by varying the concentration of polymer, as described by Pereira and co-workers [19]. The formulated polyplexes were further incubated at room temperature for 15 min and then pelleted by centrifugation (15,000 *g*, 20 min, 4 °C).

### 3.6. Gel Retardation Assay

The extent of RNA binding and condensation by the systems (CS-SA, PEI-SA, CS-SA-Lf, and PEI-SA-Lf) were investigated by agarose gel electrophoresis. Briefly, polyplexes were prepared at different N/P ratios, and, after centrifugation, 20 μL of each sample was loaded into individual wells of the 0.8% agarose gel in Tris-acetic acid buffer (40 mM Tris base, 20 mM acetic acid, and 1 mM EDTA, pH 8.0). The electrophoretic run was performed at 120 V for 50 min, and the bands corresponding to unbound RNA were visualized under ultraviolet light after staining the gels with 0.5 µg/mL greensafe (Nzytech, Lisbon, Portugal).

### 3.7. Determination of the Encapsulation Efficiency

The encapsulation efficiency (EE) was calculated by determining free RNA concentration in the supernatant recovered after particle centrifugation. The amount of unbound RNA was quantified by UV spectrophotometry (Nanophotometer) at 260 nm. Supernatant recovered from unloaded complexes (without RNA) was used as a blank. The EE was determined using the following formula: EE% = [(Total amount of RNA − amount of RNA in the supernatant)/Total amount of RNA] × 100 [47]. Data represent the mean ± standard deviations of three independent measurements for each polyplex.

### 3.8. Scanning Electron Micrograph Morphology

Scanning electron microscopy (SEM) analysis was performed to evaluate polyplexes morphology. Briefly, one drop of the solution containing the polyplexes samples was placed on the surface of cover glasses and left to dry at 37 °C overnight. Subsequently, the samples were sputter-coated with gold using a Quorum Q150R ES sputter-coater (Quorum Technologies, Lewes, UK). The SEM images were then captured with different magnifications, at an acceleration voltage of 20 kV, using a Hitachi S-3400 N scanning electron microscope (Hitachi, Tokyo, Japan).

### 3.9. Dynamic Light Scattering and Zeta Potential Analysis

The hydrodynamic particle size average (z-average), polydispersity index (PDI), and zeta potential of the polyplexes were determined using a Zetasizer Nano-ZS (Malvern Instruments, Worcestershire, UK) and examined in Zetasizer 7.11 software 2.2.5.4. Dynamic light scattering (DLS) measurements were made at 25 °C with a backward scattering angle of 173°. The polyplexes were prepared immediately before analysis, resuspended in ultrapure water, and filtered. On the other hand, zeta potential measurements of the polyplexes were performed in a zeta disposable folded capillary cell and determined by laser Doppler electrophoresis at 25 °C. The average values of size and zeta potential (mean (SEM of 10 runs for particle size) ± standard deviations) were calculated with the data obtained from three independent experiments.

### 3.10. Biological Activity

The biological activity and cytotoxicity of pre-miR-29b-loaded polyplexes were evaluated in N2a695 and RBE4 cell lines. N2a695 cells (at passages 5–27) were cultured in the following medium: 1:1 mixture of Dulbecco’s Modified Eagle Medium (DMEM) and OptiMEM supplemented with 5% (*w/v*) heat-inactivated fetal bovine serum (FBS) and 1% (*w/v*) penicillin–streptomycin. On the other hand, RBE4 cultures (at passages 7–20) were grown in the following medium: 1:1 mixture of Minimum Essential Medium (αMEM) and Ham’s F10 Nutrient Mix supplemented with 10% FBS, 1% penicillin–streptomycin, 1% L-glutamine, and 0.6% Geneticin (300 μg/mL) [58]. Cell lines were kept at 37 °C in a humidified atmosphere containing 5% CO_2_ and were subcultured regularly using trypsin-EDTA.

### 3.11. Cell Viability Assay

Cell viability in the presence of the different formulations of polyplexes was assessed, in parallel experiments, using the Cell Titer 96^®^ AQueous Non-Radioactive Cell Proliferation Assay (Promega, Madison, WI, USA), at different time points (24 and 72 h) [19]. Post-transfection, cells were incubated with a mixture of MTS/phenazine metasulfate (PMS) for 2 h according to the manufacturer instructions, at 37 °C in a humidified atmosphere containing 5% CO_2_. Following incubation, the absorbance measurements of the soluble brown formazan produced were performed in a microplate reader at 490 nm. All experiments were obtained from at least three independent samples. Cells incubated with absolute ethanol were used as positive control for cytotoxicity.

### 3.12. In Vitro Cellular Uptake—Cell Live Imaging

To investigate the cellular uptake, recombinant pre-miR-29b was labeled with the fluorescent dye, FITC. In brief, an aliquot of the FITC-DMSO solution was added in drops to the pre-miR-29b solution (10 nM). The resultant solution was kept under stirring for 3 h at room temperature. Then, the final product was incubated with 3 M NaCl and ice-cold absolute ethanol at −20 °C for 1 h. Excess FITC was removed by centrifugation at 12,000 *g* for 30 min at 4 °C. After centrifuging, the pellet was washed twice with 75% ethanol, followed by a 5 min centrifugation at 12,000 *g* (4 °C). Finally, FITC-pre-miR-29b was resuspended in OptiMEM and encapsulated with CS-SA-Lf and PEI-SA-Lf. N2a695 cells were seeded at a density of 2 × 10^4^ cells/cm^2^ in μ-Slide 8-well flat bottom imaging plates (Ibidi GmbH, Martinsried, Germany). On the following day, the medium was replaced by fresh serum-free medium, and cells were stained with Hoechst 33342^®^ nuclear probe for 20 min. Subsequently, the cells were transfected with the complexes prepared with FITC-labeled pre-miR-29b in a serum-free medium. The cells were transferred to a Zeiss LSM 710 confocal laser scanning microscope (CLSM; White Plains, NY, USA) equipped with a plane-apocromat 63×/DIC objective and processed in Zeiss Zen (SP2, 2010) and Imaris software (Bitplane, Zürich, Switzerland) to evaluate the cellular uptake. The fluorescence images were obtained at 63× amplification. Cells without the addition of complexes were imaged as control.

### 3.13. In Vitro Transfection Studies

N2a695 cells were seeded in 12-well plates at a density of 2 × 10^4^ cells/well in 1.5 mL of complete medium. When a 50 to 60% confluence was achieved, the media was replaced by a serum-free culture medium. After 12 h, pre-miR-29-loaded systems and Lipofectamine/pre-miR-29b (Lipo/pre-miR-29b) were added to the cells, at a pre-miR-29b concentration of 10 nM, and transfection was carried out for 4 h. The culture medium was replaced by fresh medium supplemented with 1% FBS and 1% antibiotic, to allow the cells to remain metabolically active, expressing human BACE1. The cells were incubated for an additional 72 h at 37 °C. Untreated cells and cells transfected with an unrelated RNA (5′-UGUGCAAAUCUAUGCAAAACUGA-3′) were used for positive controls. All transfection experiments were performed in triplicate. Total RNA was recovered with TRIzol reagent (Invitrogen, Carlsbad, CA, USA) and chloroform, further purified by isopropanol precipitation and washed with 75% ethanol. The concentration of total RNA was determined using a NanoPhotometer UV/Vis Spectrophotometer, and the integrity and quality of RNA were assessed by agarose gel electrophoresis. RNA was treated with DNase I to avoid genomic contamination. First-strand cDNA was synthesized using the RevertAid First Strand cDNA Synthesis Kit (Thermo Fisher Scientific Inc., Waltham, MA, USA) in a total volume of 20 µL containing 1 µg of total RNA, according to the manufacturer’s instructions.

### 3.14. Expression of Human BACE1 mRNA in N2a695 Cells by RT-qPCR

For quantitative analysis, RT-qPCR amplification of cDNA was performed using the Maxima^®^ SYBR Green/Fluorescein qPCR Master Mix (Thermo Fisher Scientific Inc., Waltham, MA, USA) in an IQ5 Cycler from BioRad. RT-qPCR reaction was prepared in a final volume of 20 μL containing 10 μL of Maxima^®^ SYBR Green/Fluorescein qPCR Master Mix, 1.2 μL each of 25 µM forward and reverse primers, and 1 μL of cDNA. The program was set as follows: 5 min at 95 °C, followed by 40 cycles of 30 s at 95 °C, 30 s at 62 °C, and 30 s at 72 °C. RT-qPCR efficiencies were calculated from the given slopes with MyIQ 2.0 software (BioRad, Hercules, CA, USA). The relative quantification of the BACE1 expression was based on the comparative threshold cycle (CT) method in which the amount of the target was determined to be 2-(ΔCT target—ΔCT calibrator), normalized to levels of glyceraldehyde-3-phosphate dehydrogenase (GAPDH) and relative to the untreated control cells. The primers used in these experiments were 5′-AGACGCTCAACATCCTGGTG-3′ (forward) and 5′-CCTGGGTGTAGGGCACATAC-3′ (reverse) for the amplification of human BACE (hBACE) and 5′-TGACGTGCCGCCTGGAGAAA-3′ (forward), 5′-AGTGTAGCCCAAGATGCCCTTCAG-3′ (reverse) for the amplification of GAPDH. Each sample was run in triplicate, and threshold cycle (CT) values were averaged from the triplicate. The final data were averaged from 3 separately conducted experiments.

### 3.15. Transport Across an In Vitro BBB Model

#### 3.15.1. BBB Transport Experiment

RBE4 cells were established to evaluate the ability of the polyplexes to cross the BBB model. RBE4 cells were seeded in 12-well culture insert coated with type I collagen (Polycarbonate Membrane Transwell Inserts of 1.0 mm mean pore size, 1.12 cm^2^ surface area, Corning, NY, USA). Zero point five milliliters of cell suspensions containing 2.0 × 105 cells was added to the inner (upper) chamber, inserted into the outer (lower) chamber containing 1.3 mL of the same culture medium. A cell monolayer was usually formed 3–5 days after seeding, as judged by three criteria: (1) the cells formed a confluent monolayer without visible spaces between cells under a light microscope; (2) the height of the culture medium in the inner chamber had to be at least 2 mm higher than that in the outer chamber for at least 24 h; and (3) a constant transendothelial electrical resistance (TEER) value across the cell layer was obtained [49]. TEER was used to measure the formation of tight junctions, and is indicative of the integrity of the BBB structure for studying drug delivery to the brain. The cell monolayer integrity was monitored using an EVOM Endohmchamber (Word Precision Instruments, Inc. Sarasota, FL, USA) to measure the TEER value of the in vitro BBB model. Only cell monolayers with a TEER value above 200 Ωcm^2^ were selected for the transport studies, which indicate that this system could be used as an in vitro BBB model [66]. All the permeability experiments were performed in serum-free medium at 37 °C. The polyplexes (including CS-SA/pre-miR-29b-FITC, PEI-SA/pre-miR-29b-FITC, CS-SA-Lf/pre-miR-29b-FITC, and PEI-SA-Lf/pre-miR-29b-FITC) were added into the upper chamber with the pre-miR-29b concentration of 10 nM. After 4 h, a volume of 100 µL of the content of the chambers was dispensed into a black plate. The BBB transport ratio of pre-miR-29b was determined using a spectrofluorometer (Spectramax Gemini XS, Molecular Devices LLC, USA), with the excitation and emission wavelength at 480 and 590 nm, respectively. The BBB transport ratio of pre-miR-29b was calculated as follows: Transport ratio (%) = [(Total Fluorescence in the outer chamber)/(Total Fluorescence in the inner chamber + Total Fluorescence in the outer chamber)] × 100.

#### 3.15.2. Immunofluorescence

After transfection with polyplexes, the endothelial cells from the in vitro BBB model were fixed with 4% paraformaldehyde (PFA) buffer solution for 10 min at room temperature, followed by permeabilization with PBS containing 0.1% Triton X-100 for 5 min. After fixation, the nucleus of the endothelial cells was counterstained with Hoechst 33342^®^ (1:1000) for 10 min, followed by 3 washing steps with PBS-Tween 20. Glass coverslips were mounted on slides, and the fluorescence images were acquired using a Zeiss LSM 710 laser scanning confocal microscope (Carl Zeiss Microscopy, White Plains, NY, USA), equipped with a plane-apocromat 63×/DIC objective. Images were processed and analyzed using ImageJ software. All experiments were repeated at least three times, and representative images are shown.

### 3.16. Statistical Analysis

All the experiments were repeated at least three times using independent culture preparations. Data in the figures are presented as mean ± standard error. Quantitative data were statistically analyzed by one-way analysis of variance (ANOVA), followed by pair-wise comparisons using the Fisher’s least significant difference test. A *p-*value < 0.05 was considered statistically significant. Statistical analysis was performed by using GraphPad Prism 6 software. The following nomenclature was applied: * indicate significant difference versus untreated cells, # indicates significant difference versus cells transfected with unrelated RNA control and indicate a significant difference between samples, is considered statistically significant for *p* < 0.05.

## 4. Conclusions

A simple method for the preparation of polyplexes formed by dual-functionalized (with SA and Lf) cationic polymers (CS and PEI) and dual-functionalized with SA and Lf is herein reported. In general, the dual-targeting polyplexes (CS-SA-Lf) exhibited the highest positive zeta potential values (+35 mV), intimately linked to their ability to cross the negatively charged cell membrane. These carriers displayed a typical size of circa 300 nm and higher encapsulation efficiencies (EE > 92%), further demonstrating that the protonated amine groups promoted electrostatic interactions with the negatively charged phosphate groups in RNA, neutralizing and completely surrounding the RNA biopharmaceutical. The Lf conjugated polyplexes were additionally identified as non-toxic, while in vitro bioimaging studies demonstrated their enhanced brain-targeting ability. Moreover, pre-miR-29b-loaded polyplexes induced the inhibition of BACE1 mRNA. In particular, the polyplexes of CS-SA-Lf/pre-miR-29b exhibited excellent internalization capability in neuronal cells compared with the other prepared polyplexes (CS-SA/pre-miR-29b, PEI-SA-Lf/pre-miR-29b, and PEI-SA/pre-miR-29b), and with shorter incubation times. The present in vitro study also demonstrated that CS-SA-Lf/pre-miR-29b polyplexes could efficiently cross the BBB and, thus, deliver the recombinant pre-miR-29b as a therapeutic agent to the neuronal cells. Our results suggest that CS conjugated with SA and Lf can represent a potentially promising system to effectively deliver drugs across the BBB and into the brain, aimed at the development of effective strategies to treat AD.

## Figures and Tables

**Table 1:** Characterization of chitosan–stearic acid–lactoferrin (CS-SA-Lf)/pre-miR-29b and polyethyleneimine–stearic acid–lactoferrin (PEI-SA-Lf)/pre-miR-29b polyplexes.

Polyplexes	Charge Ratio	Z-Average Diameter (nm)	Zeta Potential (mV)	EE (%)
CS-SA-Lf/pre-miR-29b	35	325.60 ± 30.99	+33.50 ± 2.31	96.35 ± 3.56
	40	432.50 ± 50.72	+37.26 ± 2.49	92.36 ± 6.70
PEI-SA-Lf/pre-miR-29b	15	289.40 ± 39.12	+10.74 ± 1.07	96.06 ± 2.81
	20	463.45 ± 47.68	+21.01 ± 3.12	94.44 ± 7.86

Abbreviations: CS, chitosan; EE, encapsulation efficiency PEI, polyethylenimine; SA, stearic acid; Lf, lactoferrin; pre-miR, microRNA percursor.

## Supplementary Materials

The following are available online at https://www.mdpi.com/1424-8247/13/10/314/s1, Figure S1: ^1^H NMR spectrum of stearic acid–chitosan (CS-SA) conjugate, Figure S2: ^1^H NMR spectrum of stearic-polyethyleneimine (PEI-SA conjugate), Figure S3: Chemical structure of chitosan (CS), Figure S4: ^1^H NMR spectrum of chitosan–stearic acid (CS-SA) conjugate coupling to lactoferrin (Lf) in D_2_O, Figure S5: ^1^H NMR spectrum of polyethyleneimine–stearic acid (PEI-SA) conjugate coupling to lactoferrin (Lf) in D_2_O, Figure S6: SDS-PAGE of the PEI-SA-Lf and CS-SA-Lf conjugates, Figure S7: Agarose gel electrophoresis of polyplexes at various N/P ratios: (A) CS-SA/RNA and (B) PEI-SA/RNA. The first lane of the gel corresponds to the molecular weight marker, and the second lane corresponds to free pre-miR-29b. The numbers in each lane indicate the N/P ratios. Each experiment was performed three times, Figure S8: SEM images for CS/PEI modified with stearic acid and lactoferrin: (A) CS-SA-Lf/RNA and (B) PEI-SA-Lf/RNA, Figure S9: Relative cell viability of N2A695 cells treated with different pre-miR-29b-loaded polyplexes at various N/P ratios for 72 h: (A) CS-based polyplexes and (B) PEI-based polyplexes.

## References

[1] Isik A.T. (2010). Late onset Alzheimer’s disease in older people. Clin. Interv. Aging.

[2] Lane C.A., Hardy J., Schott J.M. (2018). Alzheimer’s disease. Eur. J. Neurol..

[3] Meldolesi J. (2019). Alzheimer’s disease: Key developments support promising perspectives for therapy. Pharmacol. Res..

[4] Mullane K., Williams M. (2013). Alzheimer’s therapeutics: Continued clinical failures question the validity of the amyloid hypothesis-but what lies beyond?. Biochem. Pharmacol..

[5] Dorszewska J., Prendecki M., Oczkowska A., Dezor M., Kozubski W. (2016). Molecular Basis of Familial and Sporadic Alzheimer’s Disease. Curr. Alzheimer Res..

[6] Xheng H., Fridkin M., Youdim M. (2014). From single to multitarged/network therapeutics in Alzheimer’s disease. Pharmaceuticals.

[7] Ghiso J., Frangione B. (2002). Amyloidosis and Alzheimer’s disease. Adv. Drug Deliv. Rev..

[8] Provost P. (2010). Interpretation and applicability of microRNA data to the context of Alzheimer’s and age-related diseases. Aging.

[9] Schonrock N., Matamales M., Ittner L.M., Götz J. (2012). MicroRNA networks surrounding APP and amyloid-β metabolism-implications for Alzheimer’s disease. Exp. Neurol..

[10] Vassar R., Kovacs D.M., Yan R., Wong P.C. (2009). The beta-secretase enzyme BACE in health and Alzheimer’s disease: Regulation, cell biology, function, and therapeutic potential. J. Neurosci..

[11] Cole S.L., Vassar R. (2007). The Alzheimer’s disease beta-secretase enzyme, BACE1. Mol. Neurodegener..

[12] Hunt C.E., Turner A.J. (2009). Cell biology, regulation and inhibition of beta-secretase (BACE-1). FEBS J..

[13] Nilsen T.W. (2007). Mechanisms of microRNA-mediated gene regulation in animal cells. Trends Genet..

[14] Basak I., Patil K.S., Alves G., Larsen J.P., Møller S.G. (2016). microRNAs as neuroregulators, biomarkers and therapeutic agents in neurodegenerative diseases. Cell. Mol. Life Sci..

[15] Abe M., Bonini N.M. (2013). MicroRNAs and neurodegeneration: Role and impact. Trends Cell Biol..

[16] Goodall E.F., Heath P.R., Bandmann O., Kirby J., Shaw P.J. (2013). Neuronal dark matter: The emerging role of microRNAs in neurodegeneration. Front. Cell. Neurosci..

[17] Dunkel P., Chai C.L., Sperlágh B., Huleatt P.B., Mátyus P. (2012). Clinical utility of neuroprotective agents in neurodegenerative diseases: Current status of drug development for Alzheimer’s, Parkinson’s and Huntington’s diseases, and amyotrophic lateral sclerosis. Expert Opin. Investig. Drugs.

[18] Pereira P., Queiroz J.A., Figueiras A., Sousa F. (2017). Current progress on microRNAs-based therapeutics in neurodegenerative diseases. Wiley Interdiscip. Rev. RNA.

[19] Pereira P.A., Tomás J.F., Queiroz J.A., Figueiras A.R., Sousa F. (2016). Recombinant pre-miR-29b for Alzheimer’s disease therapeutics. Sci. Rep..

[20] Pardridge W.M. (2016). CSF, blood-brain barrier, and brain drug delivery. Expert Opin. Drug Deliv..

[21] Dong X. (2018). Current Strategies for Brain Drug Delivery. Theranostics.

[22] Sharma G., Sharma A.R., Lee S.S., Bhattacharya M., Nam J.S., Chakraborty C. (2019). Advances in nanocarriers enabled brain targeted drug delivery across blood brain barrier. Int. J. Pharm..

[23] Pardridge W.M. (2017). Delivery of Biologics Across the Blood-Brain Barrier with Molecular Trojan Horse Technology. BioDrugs.

[24] Löscher W., Potschka H. (2005). Role of drug efflux transporters in the brain for drug disposition and treatment of brain diseases. Prog. Neurobiol..

[25] Descamps L., Dehouck M.P., Torpier G., Cecchelli R. (1996). Receptor-mediated transcytosis of transferrin through blood-brain barrier endothelial cells. Am. J. Physiol..

[26] Visser C.C., Stevanović S., Heleen Voorwinden L., Gaillard P.J., Crommelin D.J., Danhof M., De Boer A.G. (2004). Validation of the transferrin receptor for drug targeting to brain capillary endothelial cells in vitro. J. Drug Target..

[27] Fillebeen C., Descamps L., Dehouck M.P., Fenart L., Benaïssa M., Spik G., Cecchelli R., Pierce A. (1999). Receptor-mediated transcytosis of lactoferrin through the blood-brain barrier. J. Biol. Chem..

[28] Candela P., Gosselet F., Miller F., Buee-Scherrer V., Torpier G., Cecchelli R., Fenart L. (2008). Physiological pathway for low-density lipoproteins across the blood-brain barrier: Transcytosis through brain capillary endothelial cells in vitro. Endothelium.

[29] Dehouck B., Fenart L., Dehouck M.P., Pierce A., Torpier G., Cecchelli R. (1997). A new function for the LDL receptor: Transcytosis of LDL across the blood-brain barrier. J. Cell Biol..

[30] Huwyler J., Wu D., Pardridge W.M. (1996). Brain drug delivery of small molecules using immunoliposomes. Proc. Natl. Acad. Sci. USA.

[31] Swami A., Goyal R., Tripathi S.K., Singh N., Katiyar N., Mishra A.K., Gupta K.C. (2009). Effect of homobifunctional crosslinkers on nucleic acids delivery ability of PEI nanoparticles. Int. J. Pharm..

[32] Sarvaiya J., Agrawal Y.K. (2015). Chitosan as a suitable nanocarrier material for anti-Alzheimer drug delivery. Int. J. Biol. Macromol..

[33] García-Montoya I.A., Cendón T.S., Arévalo-Gallegos S., Rascón-Cruz Q. (2012). Lactoferrin a multiple bioactive protein: An overview. Biochim. Biophys. Acta Gen. Subj..

[34] Ward P.P., Paz E., Conneely O.M. (2005). Multifunctional roles of lactoferrin: A critical overview. Cell. Mol. Life. Sci..

[35] Huang R.Q., Ke W.L., Qu Y.H., Zhu J.H., Pei Y.Y., Jiang C. (2007). Characterization of lactoferrin receptor in brain endothelial capillary cells and mouse brain. J. Biomed. Sci..

[36] Ji B., Maeda J., Higuchi M., Inoue K., Akita H., Harashima H., Suhara T. (2006). Pharmacokinetics and brain uptake of lactoferrin in rats. Life Sci..

[37] Van de Looij Y., Ginet V., Chatagner A., Toulotte A., Somm E., Hüppi P.S., Sizonenko S.V. (2014). Lactoferrin during lactation protects the immature hypoxic-ischemic rat brain. Ann. Clin. Transl. Neurol..

[38] Rousseau E., Michel P.P., Hirsch E.C. (2013). The iron-binding protein lactoferrin protects vulnerable dopamine neurons from degeneration by preserving mitochondrial calcium homeostasis. Mol. Pharmacol..

[39] Iwamaru Y., Shimizu Y., Imamura M., Murayama Y., Endo R., Tagawa Y., Ushiki-Kaku Y., Takenouchi T., Kitani H., Mohri S. (2008). Lactoferrin induces cell surface retention of prion protein and inhibits prion accumulation. J. Neurochem..

[40] Guo C., Yang Z.H., Zhang S., Chai R., Xue H., Zhang Y.H., Li J.Y., Wang Z.Y. (2017). Intranasal Lactoferrin Enhances α-Secretase-Dependent Amyloid Precursor Protein Processing via the ERK1/2-CREB and HIF-1α Pathways in an Alzheimer’s Disease Mouse Model. Neuropsychopharmacology.

[41] Onishi H., Koyama K., Sakata O., Machida Y. (2010). Preparation of chitosan/alginate/calcium complex microparticles loaded with lactoferrin and their efficacy on carrageenan-induced edema in rats. Drug Dev. Ind. Pharm..

[42] Huang R., Ke W., Han L., Liu Y., Shao K., Jiang C., Pei Y. (2010). Lactoferrin-modified nanoparticles could mediate efficient gene delivery to the brain in vivo. Brain Res. Bull..

[43] Hu P., Wang T., Xu Q., Chang Y., Tu H., Zheng Y., Zhang J., Xu Y., Yang J., Yuan H. (2013). Genotoxicity evaluation of stearic acid grafted chitosan oligosaccharide nanomicelles. Mutat. Res. Toxicol. Environ. Mutagen..

[44] Xie Y.T., Du Y.Z., Yuan H., Hu F.Q. (2012). Brain-targeting study of stearic acid-grafted chitosan micelle drug-delivery system. Int. J. Nanomed..

[45] Chuanxu Y., Shan G., Jørgen K. (2014). Folic acid conjugated chitosan for targeted delivery of siRNA to activated macrophages in vitro and in vivo. J. Mater. Chem. B.

[46] Yuan H., Lu L.J., Du Y.Z., Hu F.Q. (2011). Stearic acid-g-chitosan polymeric micelle for oral drug delivery: In vitro transport and in vivo absorption. Mol. Pharm..

[47] Jingou J., Danjun W., Li L., Chen J., Xiu Y. (2011). Preparation, evaluation, and in vitro release of folic acid conjugated O-carboxymethyl chitosan nanoparticles loaded with methotrexate. J. Appl. Polym. Sci..

[48] Pereira P., Jorge A.F., Martins R., Pais A.A., Sousa F., Figueiras A. (2012). Characterization of polyplexes involving small RNA. J. Colloid Interface Sci..

[49] Huang R., Ke W., Han L., Liu Y., Shao K., Ye L., Lou J., Jiang C., Pei Y. (2009). Brain-targeting mechanisms of lactoferrin-modified DNA-loaded nanoparticles. J. Cereb. Blood Flow Metab..

[50] Meng Q., Wang A., Hua H., Jiang Y., Wang Y., Mu H., Wu Z., Sun K. (2018). Intranasal delivery of Huperzine A to the brain using lactoferrin-conjugated N-trimethylated chitosan surface-modified PLGA nanoparticles for treatment of Alzheimer’s disease. Int. J. Nanomed..

[51] Chen Y., Zhou K., Wang R., Liu Y., Kwak Y.D., Ma T., Thompson R.C., Zhao Y., Smith L., Gasparini L. (2009). Antidiabetic drug metformin (GlucophageR) increases biogenesis of Alzheimer’s amyloid peptides via up-regulating BACE1 transcription. Proc. Natl. Acad. Sci. USA.

[52] Hildebrandt I.J., Iyer M., Wagner E., Gambhir S.S. (2003). Optical imaging of transferrin targeted PEI/DNA complexes in living subjects. Gene Ther..

[53] Zhang W., Müller K., Kessel E., Reinhard S., He D., Klein P.M., Höhn M., Rödl W., Kempter S., Wagner E. (2016). Targeted siRNA Delivery Using a Lipo-Oligoaminoamide Nanocore with an Influenza Peptide and Transferrin Shell. Adv. Healthc. Mater..

[54] Huwyler J., Froidevaux S., Roux F., Eberle A.N. (1999). Characterization of transferrin receptor in an immortalized cell line of rat brain endothelial cells, RBE4. J. Recept. Signal Transduct..

[55] Burkhart A., Skjørringe T., Johnsen K.B., Siupka P., Thomsen L.B., Nielsen M.S., Thomsen L.L., Moos T. (2016). Expression of Iron-Related Proteins at the Neurovascular Unit Supports Reduction and Reoxidation of Iron for Transport Through the Blood-Brain Barrier. Mol. Neurobiol..

[56] Roux F., Couraud P.O. (2005). Rat brain endothelial cell lines for the study of blood-brain barrier permeability and transport functions. Cell. Mol. Neurobiol..

[57] Reis K., Hälldin J., Fernaeus S., Pettersson C., Land T. (2006). NADPH oxidase inhibitor diphenyliodonium abolishes lipopolysaccharide-induced down-regulation of transferrin receptor expression in N2a and BV-2 cells. J. Neurosci. Res..

[58] Balbuena P., Li W., Ehrich M. (2011). Assessments of tight junction proteins occludin, claudin 5 and scaffold proteins ZO1 and ZO2 in endothelial cells of the rat blood-brain barrier: Cellular responses to neurotoxicants malathion and lead acetate. Neurotoxicology.

[59] Huang R., Ke W., Liu Y., Jiang C., Pei Y. (2008). The use of lactoferrin as a ligand for targeting the polyamidoamine-based gene delivery system to the brain. Biomaterials.

[60] Song Y., Du D., Li L., Xu J., Dutta P., Lin Y. (2017). In Vitro Study of Receptor-Mediated Silica Nanoparticles Delivery across Blood-Brain Barrier. ACS Appl. Mater. Interfaces.

[61] Huang F.Y., Chen W.J., Lee W.Y., Lo S.T., Lee T.W., Lo J.M. (2013). In vitro and in vivo evaluation of lactoferrin-conjugated liposomes as a novel carrier to improve the brain delivery. Int. J. Mol. Sci..

[62] Kuo Y.C., Cheng S.J. (2016). Brain targeted delivery of carmustine using solid lipid nanoparticles modified with tamoxifen and lactoferrin for antitumor proliferation. Int. J. Pharm..

[63] Agrawal M., Ajazuddin, Tripathi D.K., Saraf S., Saraf S., Antimisiaris S.G., Mourtas S., Hammarlund-Udenaes M., Alexander A. (2017). Recent advancements in liposomes targeting strategies to cross blood-brain barrier (BBB) for the treatment of Alzheimer’s disease. J. Control. Release.

[64] Pereira P., Pedro A.Q., Tomás J., Maia C.J., Queiroz J.A., Figureiras R., Sousa F. (2016). Advances in time course extracellular production of human pre-miR-29b from *Rhodovulum sulfidophilum*. Appl. Microbiol. Biotechnol..

[65] Pereira P., Sousa Â., Queiroz J., Correia I., Figueiras A., Sousa F. (2014). Purification of pre-miR-29 by arginine-affinity chromatography. J. Chromatogr. B Analyt. Technol. Biomed. Life Sci..

[66] Re F., Cambianica I., Zona C., Sesana S., Gregori M., Rigolio R., La Ferla B., Nicotra F., Forloni G., Cagnotto A. (2011). Functionalization of liposomes with ApoE-derived peptides at different density affects cellular uptake and drug transport across a blood-brain barrier model. Nanomedicine.

