# Supplementary material for: Brain-Targeted Delivery of Pre-miR-29b Using Lactoferrin-Stearic Acid-Modified-Chitosan/Polyethyleneimine Polyplexes
Source: Pharmaceuticals (Basel). 2020 Oct 15;13(10):314. doi: 10.3390/ph13100314 (PMC7602608; doi:10.3390/ph13100314)
Supplement: Supplementary file 1 [file pharmaceuticals-13-00314-s001.pdf]

Brain-targeted delivery of pre-miR-29b using lactoferrin-stearic acid-modified–Chitosan/Polyethylenimine polyplexes

Patrícia Pereira[1,2], Maria Barreira[1], Carla Cruz[1], Joana Tomás[1], Ângelo Luís[1], Augusto Q. Pedro[3], João A. Queiroz[1] and Fani Sousa[1,*]

[1] CICS-UBI – Health Sciences Research Centre, University of Beira Interior, Avenida Infante D. Henrique, Covilhã 6200-506, Portugal

[2] University of Coimbra, Centre for Mechanical Engineering, Materials and Processes, Department of Chemical Engineering, Rua Sílvio Lima-Polo II, 3030-790 Coimbra, Portugal

[3] CICECO – Aveiro Institute of Materials, Chemistry Department, University of Aveiro, Campus Universitário de Santiago, 3810-193 Aveiro, Portugal

*Corresponding author:

Dr. Fani Sousa

Phone: +351 275 329 074

Fax: +351 275 329 099

E-mail address: fani.sousa@fcsaude.ubi.pt

Postal address: Health Sciences Research Centre, University of Beira Interior, Avenida Infante D. Henrique, Covilhã, 6200-506, Portugal

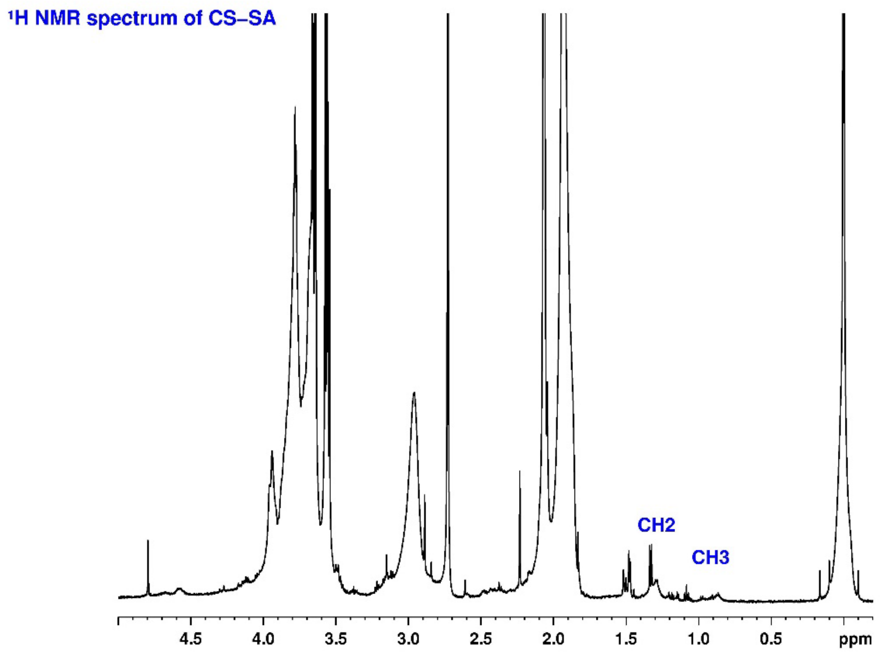

Figure S1 - ^{1}H NMR spectrum of stearic acid-chitosan (CS-SA) conjugate.

The spectrum of the CS-SA shows 3 broad peaks, which correspond to the resonance of the monosaccharide residue protons, namely a signal at δ 1.5 ppm of the carboxyl group (-$COCH_3$), the methylene group (-CH-NH-) next to hydroxyl group at 2.88 ppm and the internal CH/CH_2 groups in the chitosan ring at 3.61–4.0 ppm.

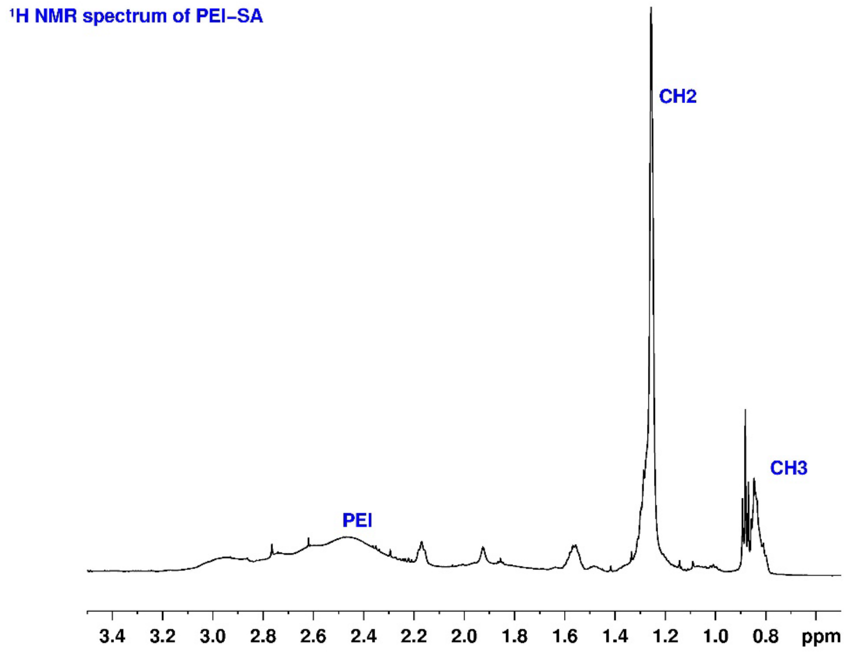

Figure S2 - ^{1}H NMR spectrum of stearic-polyethyleneimine (PEI-SA conjugate).

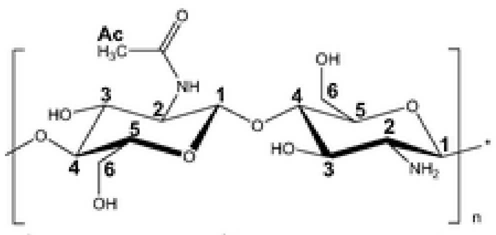

Figure S3 - Chemical structure of chitosan (CS).

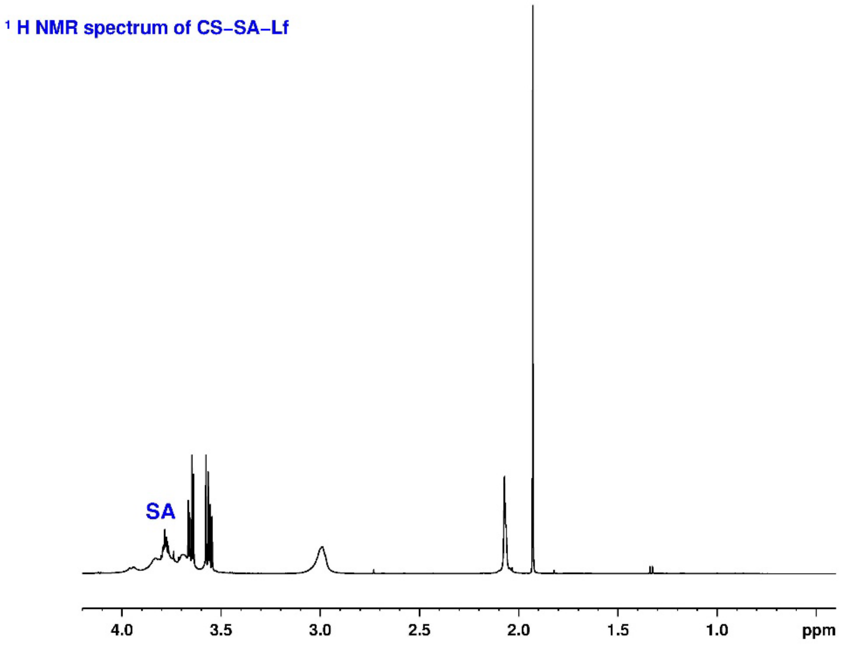

Figure S4 - ^{1}H NMR spectrum of chitosan-stearic acid (CS-SA) conjugate coupling to lactoferrin (Lf) in D_2O.

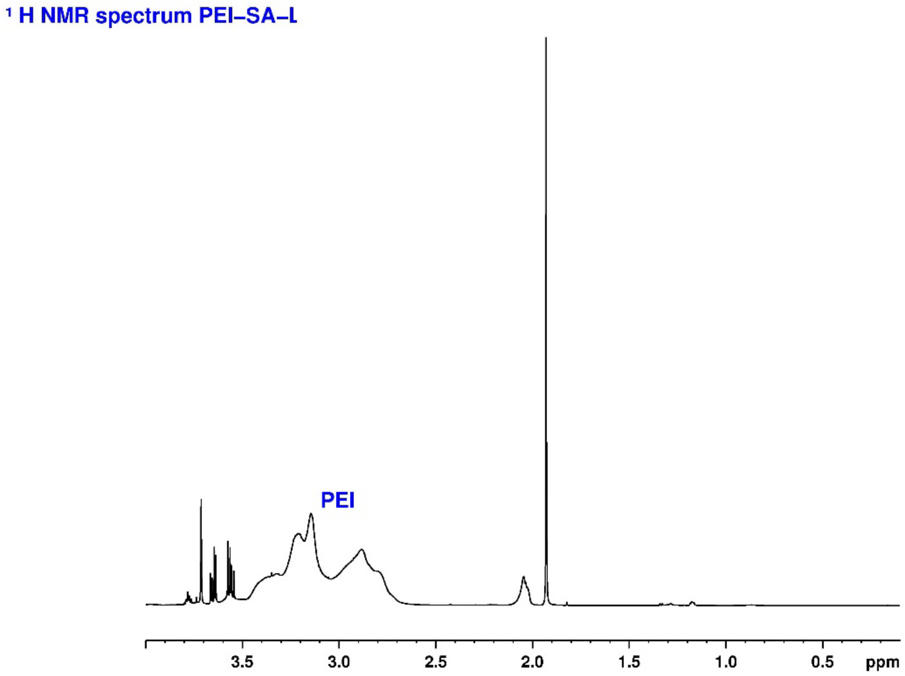

Figure S5 - ^{1}H NMR spectrum of polyethyleneimine-stearic acid (PEI-SA) conjugate coupling to lactoferrin (Lf) in D_2O.

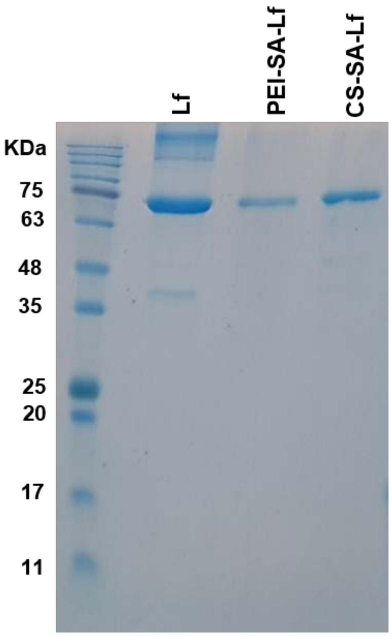

Figure S6 - SDS-PAGE of the PEI-SA-Lf and CS-SA-Lf conjugates.

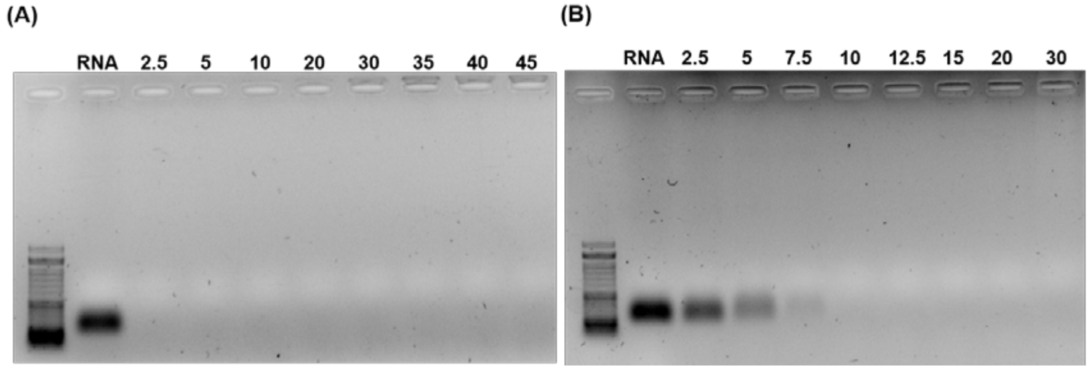

Figure S7 - Agarose gel electrophoresis of polyplexes at various N/P ratios: **(A)** CS-SA/RNA and **(B)** PEI-SA/RNA. The first lane of the gel corresponds to the molecular weight marker and the second lane corresponds to free pre-miR-29b. The numbers in each lane indicate the N/P ratios. Each experiment was performed three times.

According to the electrophoresis analysis shown in Figure S7, it was possible to observe that the RNA complexation by CS-SA conjugates starts at very low ratios, namely N/P=2.5, suggesting that RNA is fully complexed. In turn, in the case of the PEI-SA/RNA polyplexes, it was observed that the lanes corresponding to the N/P ratios of 2.5, 5 and 7.5 contain free RNA. However, above the N/P ratio of 7.5,

the band corresponding to free RNA was not observed, indicating that the RNA was efficiently conjugated with the PEI-SA.

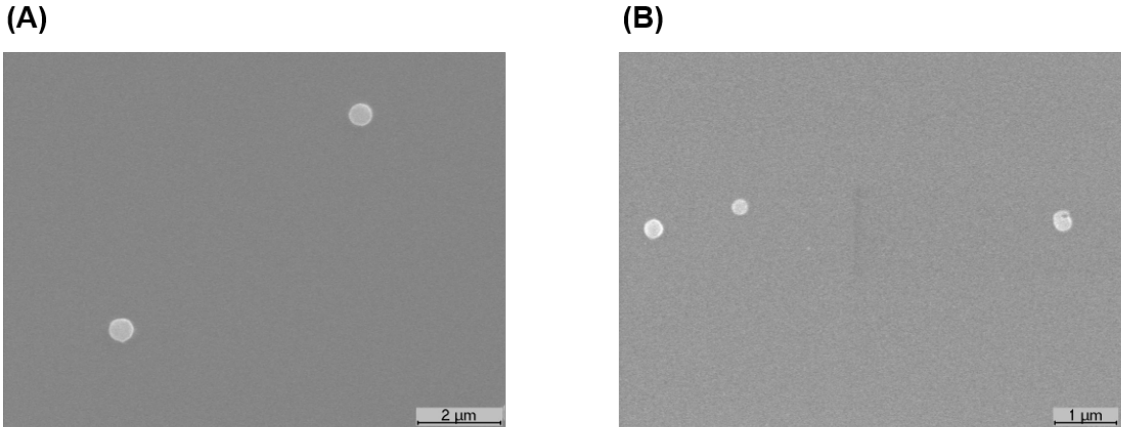

Figure S8 – SEM images for CS/PEI modified with stearic acid and lactoferrin: **(A)** CS-SA-Lf/RNA and **(B)** PEI-SA-Lf/RNA.

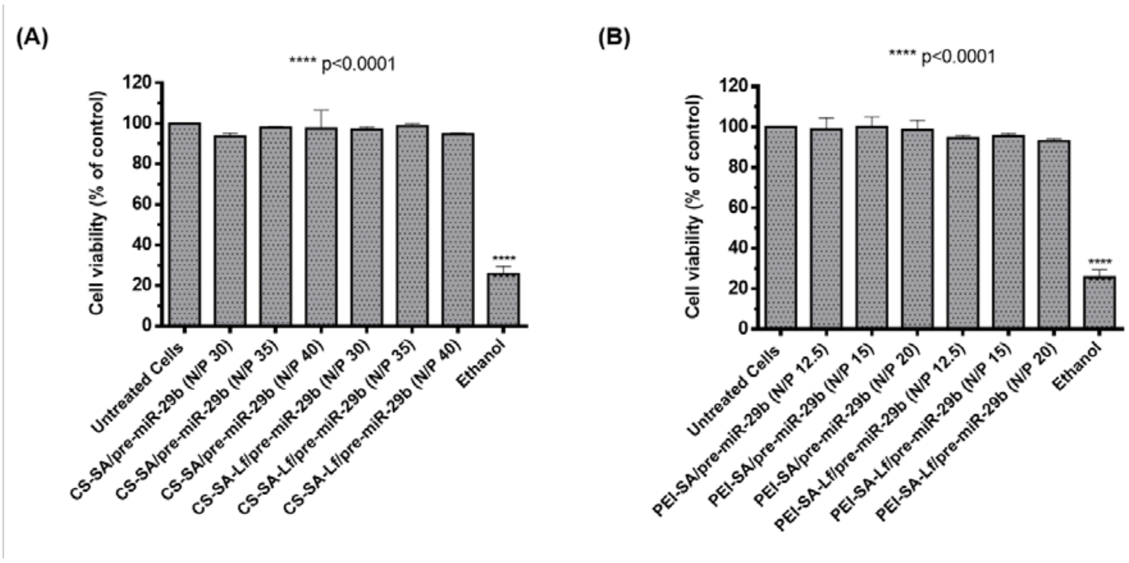

Figure S9 – Relative cell viability of N2A695 cells treated with different pre-miR-29b-loaded polyplexes at various N/P ratios for 72 hours: **(A)** CS-based polyplexes and **(B)** PEI-based polyplexes.

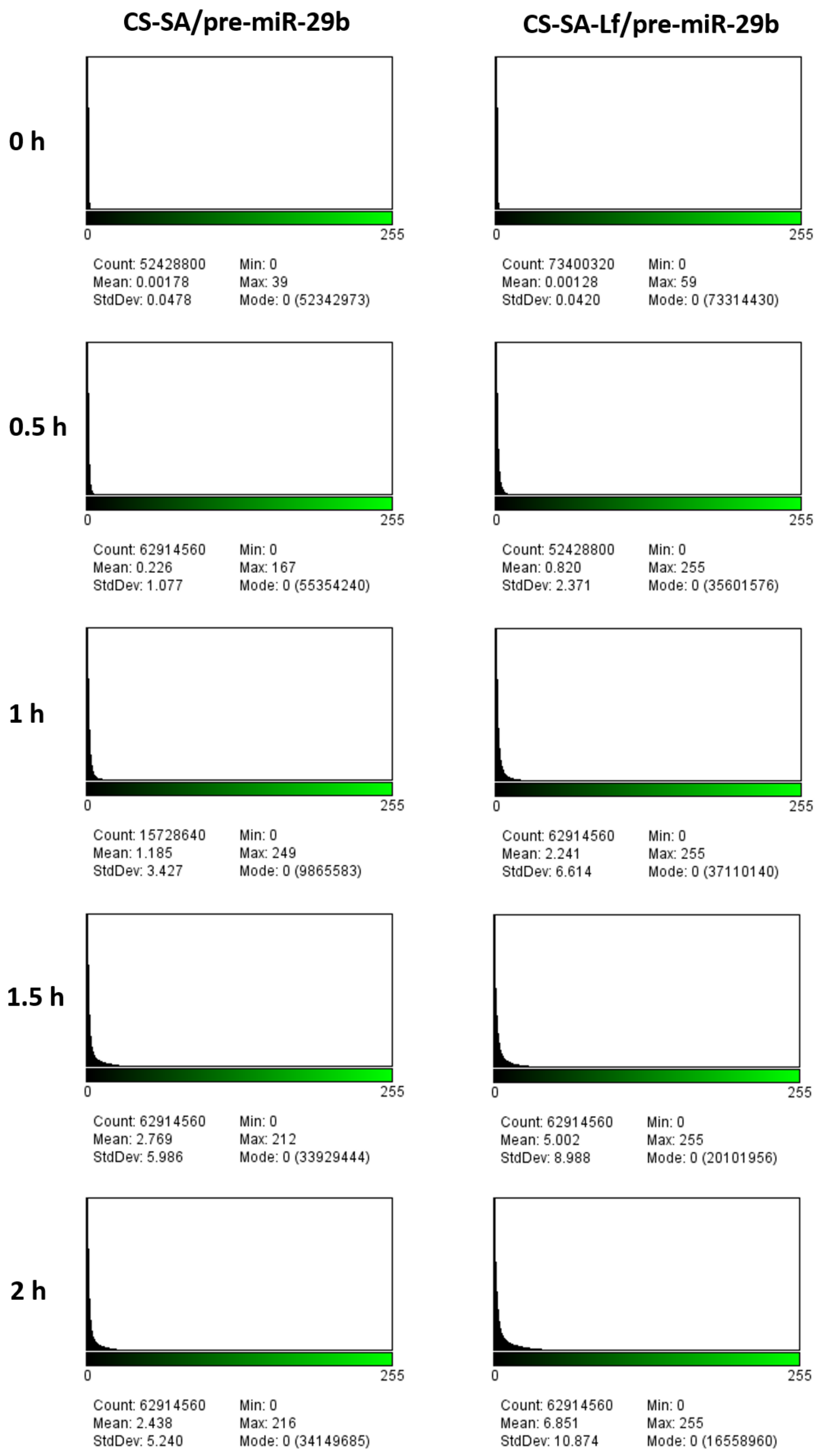
CS-SA/pre-miR-29b
CS-SA-Lf/pre-miR-29b
0 h
0.5 h
1 h
1.5 h
2 h
0
255
Count: 52428800 Min: 0
Mean: 0.00178 Max: 39
StdDev: 0.0478 Mode: 0 (52342973)
Count: 73400320 Min: 0
Mean: 0.00128 Max: 59
StdDev: 0.0420 Mode: 0 (73314430)
Count: 62914560 Min: 0
Mean: 0.226 Max: 167
StdDev: 1.077 Mode: 0 (55354240)
Count: 52428800 Min: 0
Mean: 0.820 Max: 255
StdDev: 2.371 Mode: 0 (35601576)
Count: 15728640 Min: 0
Mean: 1.185 Max: 249
StdDev: 3.427 Mode: 0 (9865583)
Count: 62914560 Min: 0
Mean: 2.241 Max: 255
StdDev: 6.614 Mode: 0 (37110140)
Count: 62914560 Min: 0
Mean: 2.769 Max: 212
StdDev: 5.986 Mode: 0 (33929444)
Count: 62914560 Min: 0
Mean: 5.002 Max: 255
StdDev: 8.988 Mode: 0 (20101956)
Count: 62914560 Min: 0
Mean: 2.438 Max: 216
StdDev: 5.240 Mode: 0 (34149685)
Count: 62914560 Min: 0
Mean: 6.851 Max: 255
StdDev: 10.874 Mode: 0 (16558960)

Figure S10 – Histogram of FITC-fluorescence intensity obtained by confocal microscopy on N2a695 cells.
